# Haemostatic changes associated with fluid resuscitation in canine parvoviral enteritis

**DOI:** 10.4102/jsava.v91i0.2005

**Published:** 2020-07-29

**Authors:** Zandri Whitehead, Amelia Goddard, Willem J. Botha, Paolo Pazzi

**Affiliations:** 1 Department of Companion Animal Clinical Studies, Faculty of Veterinary Science, University of Pretoria, Pretoria, South Africa; 2 Tygerberg Animal Hospital, Cape Town, South Africa; 3 Panorama Veterinary Hospital and Specialist Centre, Cape Town, South Africa

**Keywords:** Parvoviral enteritis, haemostasis, thromboelastography, veterinary science, fluid resuscitation

## Abstract

The haemostatic status of dogs with canine parvovirus (CPV) enteritis, within 24 h of admission after initial fluid administration, has been described previously, but the haemostatic status at admission and after standard fluid resuscitation, as well as after initial fluid redistribution, has not been investigated previously. The objective of this study was to characterise the haemostatic status at admission and describe the effect of crystalloid fluid resuscitation on haemostatic variables in dogs with CPV enteritis. Twenty-seven client-owned, hospitalised dogs with confirmed natural CPV infection and 15 healthy age-matched controls were included in a prospective, observational clinical study. The volume of resuscitation fluid, haematocrit (HCT), platelet count, thromboelastography (TEG) variables, antithrombin (AT) activity, fibrinogen- and C-reactive protein (CRP) concentrations were measured in all dogs at admission, after fluid resuscitation and, in 10 dogs, after receiving an additional 3 hours of maintenance-rate crystalloid fluids. For the CPV group at admission, the median TEG reaction time (R) and maximum amplitude (MA) or clot strength, as well as the median HCT, fibrinogen and CRP concentrations, were significantly increased compared to the controls. After fluid resuscitation, median R was significantly shorter, MA significantly increased and HCT and AT activity significantly decreased compared to admission values. The haemostatic variables remained unchanged after 3 h of maintenance-rate crystalloid therapy. The increased clot strength present in dogs with CPV enteritis at admission was exacerbated after fluid resuscitation and persisted for hours after large-volume crystalloid fluid administration.

## Introduction

Canine parvovirus (CPV) is a small, non-enveloped single-stranded deoxyribonucleic acid (DNA) virus that preferentially infects tissues with rapid cell turnover, including the intestinal epithelium and bone marrow (Parrish 1995; Pollock & Coyne 1993; Smith-Carr, Macintire & Swango 1997). Intestinal epithelial damage is associated with the risk of bacterial translocation, the development of systemic inflammation, septic shock and death (Goddard & Leisewitz 2010; Otto, Drobatz & Soter 1997). Endotoxin and pro-inflammatory cytokines, which are integral to the pathophysiology of CPV enteritis, are potent mediators of inflammation and can lead to systemic activation of haemostasis (Otto et al. 1997; Weiss & Rashid 1998).

Viscoelastic methods of coagulation testing such as thromboelastography (TEG) and thromboelastometry (TEM) combine the evaluation of the traditional plasma components with the cellular components of haemostasis and allow for the evaluation of clot formation, clot strength and fibrinolysis (Donahue & Otto 2005; Mallet & Cox 1992; Wiinberg et al. 2005).

Hypercoagulability, based on increased maximum amplitude (MA) or clot strength, a TEG variable, increased fibrinogen concentration and decreased antithrombin (AT) activity, has been reported in dogs with CPV enteritis, within 24 hours of admission after initial fluid administration (Otto et al. 2000). Hypercoagulability, characterised by decreased reaction time (R) and clot formation time (K), and increased α-angle and MA, has also been demonstrated in adult dogs with protein-losing enteropathy (Goodwin et al. 2011). The mechanism of hypercoagulability in CPV enteritis is most likely multifactorial, including an increased fibrinogen concentration secondary to the inflammation associated with CPV enteritis; loss of AT through the gastrointestinal tract secondary to loss of gastrointestinal wall integrity and severe diarrhoea; and an endotoxin-mediated procoagulant effect on vascular endothelial cells (Goddard & Leisewitz 2010; Otto et al. 2000). In adult dogs with protein-losing enteropathy, the hypercoagulability could not be attributed solely to intestinal loss of AT, but disease-associated inflammation was proposed to result in an acute phase response and hyperfibrinogenaemia, which alter TEG variables such as K, α-angle and MA (Donahue & Otto 2005; Goodwin et al. 2011). Sepsis, as is commonly seen in CPV enteritis, is associated with the development of thrombosis in a small subset of dogs, while thromboemboli have been described in dogs with protein-losing enteropathy (DeLaforcade et al. 2019; Jacinto et al. 2017). Increased clot strength and decreased AT activity are likely to contribute to the thrombosis seen in these patients.

The initial standard therapy of dogs with CPV enteritis is the administration of intravenous fluids, usually crystalloids, to correct hypovolaemia and dehydration. Decreased haematocrit (HCT) due to anaemia or haemodilution with crystalloid fluid supplementation has been reported to result in a relative increased clot strength, or MA, while a high HCT generally results in a relatively decreased MA (Bochsen et al. 2011; McMichael et al. 2011, 2014; Nagler et al. 2013; Smith et al. 2012). Dogs with CPV enteritis may present with relative haemoconcentration due to dehydration, but the HCT may decrease as a result of intestinal haemorrhage or intravenous fluid therapy (Hoskins 1997). Moreover, the administered intravenous fluid perfuses previously hypoperfused intestines, resulting in ischaemia-reperfusion injury and the release of pro-inflammatory mediators and, by extension, tissue factor, which is the primary physiological initiator of haemostasis (DelGiudice & White 2009; Krentz & Allen 2017). Fluid administration could thus influence the results of viscoelastic methods of coagulation testing, such as TEG, in dogs with CPV enteritis.

An *in vitro* canine study evaluating haemodilution of citrated blood reported no statistically significant differences for TEM variables after 10% and 25% dilution with 0.9% saline (Falco et al. 2012). Another study evaluating *in vitro* dilution of canine whole blood with lactated ringer solution (LRS), using kaolin-activated TEG, found no significant difference in R, at 33% dilution, but reported a reduced α-angle at 67% dilution. The MA was significantly decreased at both dilutions (Morris et al. 2016). These findings are in contrast to the studies by Bochsen, McMichael, Smith, Nagler and McMichael. An *in vivo* study that assessed the effect of clinically relevant doses of resuscitative fluids in dogs, using TEM, found that maximum clot firmness decreased after treatment with all fluid types, possibly the result of a dilutional effect on platelets and fibrinogen (Seshia et al. 2018).

A rapid and large increase in blood volume occurs with rapid administration of isotonic crystalloid fluids, and while the blood volume decreases rapidly after the completion of fluid infusion (the result of redistribution to the interstitium), nearly 30% of the infused fluid remains in the intravascular space 30 min after the cessation of fluid therapy (Silverstein et al. 2005). The residual volume of infused fluid decreases in a linear fashion, reaching approximately 18% of infused volume at 4 h.

The haemostatic changes in dogs with CPV enteritis from presentation until after fluid resuscitation, as well as after the initial fluid redistribution, has not been described. The aims of this study were to describe the haemostatic changes in dogs with CPV enteritis at admission compared to controls, and evaluate the effects of crystalloid fluid therapy on haemostatic variables in dogs with CPV enteritis. An additional aim was to determine if the effect of crystalloid fluid resuscitation on haemostasis outlasts fluid redistribution. We hypothesised that (1) dogs with CPV enteritis would have a higher MA at admission compared to control dogs and (2) that fluid resuscitation would result in a further increase in MA that outlasts the expected fluid redistribution.

## Research methods and design

### Study design and selection of animals

Client-owned dogs presenting to the Onderstepoort Veterinary Academic Hospital (OVAH) between January 2016 and January 2017 with naturally occurring CPV enteritis, and healthy control dogs, were enrolled in a prospective, observational study. Dogs in both the CPV and control groups were between 8 weeks and 9 months of age and weighed more than 4.5 kg. Control dogs were presented for vaccination, ovariohysterectomy or castration and were deemed healthy based on the absence of vomiting or diarrhoea in the preceding 14 days, having had no contact with dogs affected by CPV enteritis, a normal clinical examination and a peripheral blood smear negative for blood-borne parasites. Dogs presenting with clinical signs associated with CPV enteritis such as lethargy, anorexia, vomiting, diarrhoea, dehydration and/or hypovolaemic shock were tested for CPV. Dogs that tested positive using CPV antigen ELISA (Anigen Rapid CPV Ag test kit, BioNote Inc., Gyeonggi-do, Republic of Korea or IDEXX Canine Parvovirus Antigen test kit, IDEXX Laboratories Inc., Maine, United States [US]), were preliminarily diagnosed with CPV enteritis and were enrolled in the CPV group. Eligible cases were only enrolled if they were admitted for in-hospital treatment and had not received any treatment prior to admission. Infection with CPV was confirmed by faecal electron microscopy. Dogs that received any medication known to interfere with normal haemostasis – such as corticosteroids, non-steroidal anti-inflammatory drugs or anticoagulant dugs, during hospitalisation or one month prior to hospitalisation – and dogs that had previously received a transfusion of any blood products were excluded from both the control and CPV groups. Dogs were deemed to be in hypovolaemic shock if they showed clinical signs indicative of abnormalities in perfusion such as tachycardia (> 140 beats per minute), peripheral vasoconstriction with cool extremities, hypothermia (< 37.5 °C), a prolonged capillary refill time (> 2 s), poor peripheral pulse quality and mental dullness (Mazzaferro & Powell 2013). The degree of dehydration was estimated based on the presence of tacky or dry mucous membranes (at least 5% dehydration), increased skin tenting (at least 7% dehydration), a weak pulse and tachycardia (at least 10% dehydration), sunken eyes, cold extremities or hypothermia (at least 12% dehydration) (Mazzaferro & Powell 2013).

Canine parvovirus-affected dogs were cared for according to the standard treatment guidelines of the institution and all dogs received 1 mg/kg maropitant subcutaneously (SC) once daily, 20 mg/kg ampicillin IV every 8 h and 15 mg/kg metronidazole IV every 12 h, during the duration of data collection. Fentanyl constant rate infusion (CRI) was administered at 3 μg/kg/h in dogs with severe abdominal discomfort. Any other required treatment was administered after the last blood sample was collected. The control group did not receive any treatment. None of the dogs received any drugs known to affect haemostasis during the resuscitation period, and 0.9% NaCl without additional heparin was used to flush IV catheters.

### Sample collection and fluid resuscitation

Clinical parameters were recorded for each dog in the control and CPV groups, and percentage dehydration or presence of hypovolaemic shock was recorded in the CPV group. The first blood collection was performed at admission, prior to any treatment or procedures for the control and CPV groups. Blood was again collected in the CPV group after fluid resuscitation (hypovolaemic dogs) or 90 min – 110 min of fluid therapy (dehydrated dogs). A third sample was collected from 10 randomly selected dogs in the CPV group, by blinded draw of a card (A or B) from an envelope, 3 h after fluid resuscitation while on a 10 mL/kg/h CRI of LRS. Blood was collected via jugular venipuncture, using a 21G needle into evacuated serum and citrate tubes in the recommended order (Goggs et al. 2014).

In dogs with CPV that presented in hypovolaemic shock, intravenous LRS (Fresenius Kabi, Pty Ltd., Midrand, South Africa) boluses of 10 mL/kg bodyweight were given over 10 s – 60 s, and repeated every 15 min, until the normalisation of five out of seven clinical variables (alert mentation, a pink mucous membrane colour, a capillary refill time of 1 s – 2 s, a heart rate of 70–140 beats per min, a strong pulse quality, a respiratory rate of 10–30 breaths per min and a temperature of 37.5 °C – 39.5 °C) was achieved (defined as the completion of fluid resuscitation). In addition, if dehydration was identified in these hypovolaemic dogs, rehydration fluids were initiated at the same time, which entailed the replacement of the estimated percentage dehydration (over 24 h) in addition to maintenance fluid requirements (60 mL/kg/day – 80 mL/kg/day) using LRS. In the hypovolaemic group, blood collection was repeated within 15 min of fluid resuscitation completion, while still on rehydration fluids. For the dehydrated dogs with CPV without signs of hypovolaemia, rehydration entailed the same fluid plan as dehydrated hypovolaemic dogs (excluding the fluid boluses). Repeat blood collection was performed at a pre-determined time of 90 min – 110 min of fluid therapy in all of the dehydrated dogs, while still on rehydration fluids, to be of a similar time period to what was expected for fluid resuscitation in the hypovolaemic dogs. Blood collection was not repeated in the control group.

To assess the effect of initial fluid redistribution after the initial fluid resuscitation of 90 min – 110 min of fluid therapy, a blood sample was also collected as previously described, in 10 randomly selected dogs with CPV, 3 h after an additional 10 mL/kg (approximating maintenance-rate fluids and for standardisation across hydration status) of LRS (defined as the CRI group).

### Laboratory analysis

Blood samples from admission (CPV and control groups) and after fluid resuscitation (dehydrated and hypovolaemic CPV groups) were analysed. Citrated whole blood was kept at room temperature for 30 min before a tissue factor-activated TEG analysis using the TEG 5000 Thrombelastograph Hemostasis Analyzer system (Haemonetics Corporation, Massachusetts, US) was performed. The principal investigator performed all of the TEG analyses. Machine blood counts (Advia 2120, Siemens, Germany) to determine HCT and platelet count were performed on citrated whole blood at the time of each TEG assay. The HCT and platelet counts were corrected for the dilutional effect of the citrate anticoagulant (Dumont et al. 2017). The TEG variables recorded included R (reaction time), K (clot formation time), α-angle (rapidity of fibrin build-up and cross-linking) and MA. Citrated plasma and serum were frozen at −80 °C for the measurement of fibrinogen and C-reactive protein (CRP) concentrations, as well as AT activity as a batch at the end of the study period. Fibrinogen concentration was measured using the Clauss method according to the manufacturer’s instructions (ACL Elite analyzer, Instrumentation Laboratory, Massachusetts, US). Antithrombin was measured using a thrombin-dependent chromogenic substrate assay (Cobas Integra 400 plus analyzer, Roche Diagnostics, Indiana, US). A pooled control sample (presumed AT activity of 100%) was also run and the patient’s AT activity was normalised against the pooled control sample. The C-reactive protein concentration was measured using the Gentian Canine CRP Immunoassay (Gentian Diagnostics AS, Moss, Norway).

### Statistical methods

Statistical analyses were performed using a commercial software package (SPSS Statistics Software version 24, IBM Corp, Armonk, NY, US). Hypovolaemic and dehydrated dogs (subgroups) were grouped together as the CPV group. The Shapiro-Wilk test was used to assess the data for normality. The Chi-Square test was used to compare sex proportions and the Mann-Whitney *U* test for age and weight differences between groups and subgroups. The Mann-Whitney *U* test was used to determine differences in measured variables between CPV and control groups at admission. The Kruskal-Wallis test was used to determine differences between dehydrated, hypovolaemic and control dogs at admission, with post-hoc Bonferroni correction. The Wilcoxon signed-rank test was used to determine differences between variables at admission and after fluid resuscitation in the CPV group, and in the dehydrated and hypovolaemic subgroups. The Mann-Whitney *U* test was used to determine differences between dehydrated and hypovolaemic dogs after fluid resuscitation.

After fluid resuscitation, the dogs in the CRI group were evaluated as a whole, and subgroup analysis for the dehydrated and hypovolaemic dogs was performed. The Wilcoxon signed-rank test was also used to determine differences between variables from fluid resuscitation until after the CRI of LRS in the 10 randomly selected CPV dogs, and in the dehydrated and hypovolaemic subgroups. The Mann-Whitney *U* test was used to determine differences between dehydrated and hypovolaemic dogs after the CRI of LRS infusion. Correlations were determined using Spearman’s rank correlation coefficient for the CPV group at admission and after fluid resuscitation; subgroup analysis was also performed. A *p*-value of less than 0.05 was considered significant. Data are presented as median and interquartile range (IQR), unless specified otherwise.

### Ethical consideration

The study was reviewed and approved by the University of Pretoria Animal Ethics Committee (AEC number V092-15). Informed written consent was obtained from all owners.

## Results

### Animals

Twenty-eight dogs with naturally occurring CPV enteritis and 15 healthy control dogs were enrolled in the study. One dog was excluded after a negative result on faecal electron microscopy. Canine parvovirus-infected dogs consisted of 16 dogs that presented with hypovolaemic shock and 11 with dehydration. All dogs with hypovolaemic shock were also dehydrated and therefore received fluid boluses as well as concurrent rehydration and maintenance rate fluids. The signalment of the CPV and control groups is included in Table 1. No significant differences were identified between groups or subgroups for age, sex or weight. Percentage dehydration for all dogs, except one, ranged between < 5% and 7%, with only one dog deemed 10% dehydrated. No significant difference was found in the degree of dehydration between the hypovolaemic dogs and dehydrated dogs.

**TABLE 1:** Signalment of dogs included in the canine parvovirus group and control group.

Variable	Control dogs (*N* = 15)	CPV dogs (*N* = 27)
**Age (months)**		
Median	3	5
IQR	2–7	2–6
**Sex**		
Male	7	14
Female	8	13
**Weight (kg)**		
Median	9.8	10.4
IQR	7.2–14.2	5.6–14.8

CPV, canine parvovirus; IQR, interquartile range.

The principal investigator collected all of the samples. There was no significant difference in the timing of sampling between the dehydrated and hypovolaemic CPV subgroups. The median volume of fluid administered between the first and second blood collection for the hypovolaemic group was 286 mL (246 mL – 470 mL) and was significantly higher compared to the 54 mL (51 mL – 116 mL) for the dehydrated group (*p* < 0.001); equivalent to 26.5 mL/kg (19.8 mL – 39.0 mL) and 10 mL/kg (8.1 mL – 11.3 mL), respectively.

Of the 10 dogs randomly selected to receive a CRI of 10 mL/kg of LRS over 3 h, seven were from the group that presented with initial hypovolaemia and three with dehydration only. No significant difference was identified for age, sex or weight between these two subgroups.

### Thromboelastography variables at admission, after fluid resuscitation and after constant rate infusion

Table 2 contains a summary of the descriptive data of the TEG variables. At admission, the median R and K were significantly longer (*p* < 0.001 and *p* = 0.020, respectively), the α-angle was significantly smaller (*p* = 0.037) and the MA significantly larger (*p* < 0.001) in the CPV group compared to the control group. The hypovolaemic group had significantly longer R and K, and larger MA compared to the control group (*p* = 0.001, *p* = 0.033 and *p* = 0.001, respectively). The dehydrated group only showed a larger MA compared to the control group (*p* = 0.018). No significant differences were found between the hypovolaemic and the dehydrated groups at admission.

**TABLE 2:** Haemostatic and inflammatory variables in the canine parvovirus group, canine parvovirus subgroups and control group at admission and post fluid resuscitation.

Variable	Reference interval for adult dogs	Control dogs (*N* = 15)	Admission			Post fluid resuscitation		
			CPV (*N* = 27)	Hypovolaemic CPV (*N* = 16)	Dehydrated CPV (*N* = 11)	CPV (*N* = 27)	Hypovolaemic CPV (*N* = 16)	Dehydrated CPV (*N* = 11)
**HCT (L/L)**								
Median	0.37–0.55	0.35	0.44*	0.44	0.43	0.37********	0.39***********	0.35****
IQR	-	0.22–0.43	0.35–0.48	0.35–0.52	0.35–0.48	0.34–0.42	0.32–0.42	0.33–0.42
**Platelet count (x 10^9^/L)**								
Median	200–500	325	296	296	292	301******	309	301
IQR	-	276–463	230–427	222–477	253–402	235–383	206–409	235–318
**R (min)**								
Median	2.0–11.0	4.9	8.9**	9.2****	7.9	7.4******	7.6	7.3
IQR	-	2.4–7.6	6.9–10.4	7.4–10.9	5.7–9.6	5.8–9.3	6.2–10.1	5.4–7.9
**K (min)**								
Median	1.0–5.0	1.4	2.2*	2.5***	2.0	2.0******	2.0	1.8
IQR	-	0.8–2.6	1.8–3.1	2.1–3.3	1.5–2.7	1.4–2.6	1.5–3.1	1.2–2.3
**α-angle (degrees)**								
Median	34.0–77.0	70.7	61.5*	58.4	62.6	65.3******	64.8	65.3
IQR	-	56.6–78.4	53.2–65.9	54.3–65.5	53.2–67.5	60.5–71.2	53.5–70.4	61.8–73.8
**MA (mm)**								
Median	42.0–7.1	67.0	77.0**	77.2****	76.0***	78.3*******	80.8**********	78.1
IQR	-	56.3–70.1	72.1–80.3	73.4–80.0	69.2–81.7	75.9–82.2	76.2–82.7	75.9–80.1
**AT (% activity)**								
Median	80–100	80.2	81.2	82.1	80.8	76.3********	74.7**********	82.1*********
IQR	-	71.5–88.9	77.2–88.6	77.4–90.1	76.3–88.6	70.3–83.0	70.2–79.3	71.5–83.3
**Fibrinogen (mg/dL)**								
Median	200–300	276	> 700**	> 700*****	> 700*****	> 700*****	> 700	> 700
IQR	-	250–360	-	-	-	-	-	-
**CRP (mg/L)**								
Median	0–10.0	6.43	135.10**	135.60*****	133.40*****	117.9******	96.4*********	88.8**********
IQR	-	4.74–12.76	98.95–155.07	105.00–155.00	76.00–155.50	103.38–148.23	75.4–136.6	52.8–106.9

Note: CPV subgroups at admission and after fluid resuscitation.

CPV, canine parvovirus; IQR, interquartile range; HCT, haematocrit; R, reaction time; K, clot formation time; MA, maximum amplitude; AT, antithrombin; α-angle, rapidity of fibrin build-up and cross-linking.

Significant difference: *, *p* < 0.05; **, *p* < 0.001, between control and CPV group at admission.

Significant difference: ***, *p* < 0.05; ****, *p =* 0.001; ^*****, *p* < 0.001 between control and CPV subgroups at admission.

Significant difference: ******, *p* < 0.05; *******, *p* < 0.01; ********, *p* < 0.001 between CPV group at admission and after fluid resuscitation.

Significant difference: *********, *p* < 0.05; **********, *p* < 0.01; ***********, *p* < 0.001 within CPV subgroups at admission and after fluid resuscitation.

After fluid resuscitation, the median R and K were significantly shorter (*p* = 0.028 and *p* = 0.020, respectively), and the α-angle and MA significantly larger (*p* = 0.014 and *p* = 0.004, respectively) in the CPV group compared to their values at admission. In the hypovolaemic group, the MA was significantly larger (*p* = 0.007), while no significant TEG variable changes were seen in the dehydrated group from admission to after fluid resuscitation. No significant differences were found between the hypovolaemic group and the dehydrated group after fluid resuscitation.

No significant changes in the TEG variables were seen in the CPV group that received the CRI from the end of the initial fluid resuscitation up until 3 h of the CRI at 10 mL/kg, or between the hypovolaemic and dehydrated subgroups that received the CRI (Table 3).

**TABLE 3:** Haemostatic and inflammatory variables in the 10 canine parvovirus dogs at the end of the initial fluid resuscitation and after 3 h of a lactated ringer solution constant rate infusion. No significant differences were found between canine parvovirus groups or subgroups at fluid resuscitation or after 3 h of a lactated ringer solution constant rate infusion.

Variable	End of initial fluid resuscitation			Post CRI (3 hours)		
	CPV (*N* = 10)	Hypovolaemic CPV (*N* = 7)	Dehydrated CPV (*N* = 3)	CPV (*N* = 10)	Hypovolaemic CPV (*N* = 7)	Dehydrated CPV (*N* = 3)
**HCT (L/L)**						
Median	0.35	0.37	0.33	0.34	0.36	0.32
IQR	0.31–0.39	0.29–0.40	0.32 – -	0.30–0.39	0.25–0.40	0.31– -
**Platelet count (x 10^9^/L)**						
Median	305	295	315	296	292	301
IQR	246–330	250–351	235– -	249–367	249–410	202– -
**R (min)**						
Median	6.9	6.0	7.7	7.6	7.4	7.8
IQR	5.8–11.6	5.6–11.5	5.8– -	6.7–10.2	6.2–9.5	7.2– -
**K (min)**						
Median	1.7	1.6	1.8	1.8	1.8	1.7
IQR	1.2–3.1	1.2–4.0	1.2– -	1.6–3.7	1.6–3.6	1.6– -
**α-angle (degrees)**						
Median	69.4	70.4	68.3	68.9	68.5	69.2
IQR	58.0–73.3	49.7–73.1	60.7– -	52.0–70.1	52.4–70.8	50.9– -
**MA (mm)**						
Median	79.2	80.5	78.3	81	82.8	79.1
IQR	77.8–82.6	77.8–82.7	75.9– -	77.1–83.1	76.4–83.1	77.9– -
**AT (% activity)**						
Median	76.7	77.5	68.8	75.1	77.2	61.6
IQR	68.4–80.0	72.9–79.3	59.8– -	64.4–82.3	68.0–81.6	60.0– -

CPV, canine parvovirus; IQR, interquartile range; HCT, haematocrit; R, reaction time; K, clot formation time; MA, maximum amplitude; AT, antithrombin; α-angle, rapidity of fibrin build-up and cross-linking.

### Other haemostatic and inflammatory variables at admission, after fluid resuscitation and after constant rate infusion

Table 2 contains a summary of the descriptive data. The median HCT at admission was significantly higher (*p* = 0.016) in the CPV group compared to the controls; however, the platelet count was similar between groups. Compared to the controls, the median fibrinogen and CRP concentrations were significantly higher in the CPV group (*p* < 0.001 for both). The median AT activity was similar between groups. Both the hypovolaemic and dehydrated groups had significantly higher fibrinogen and CRP concentrations than the control group (*p* < 0.001). No significant differences were found between the hypovolaemic and dehydrated groups at admission.

After fluid resuscitation, for the CPV group the median HCT was significantly lower (*p* < 0.001) and the platelet count significantly higher (*p* = 0.034) compared to their values at admission. The median CRP concentration and AT activity were significantly lower compared to their values at admission (*p* = 0.001 and *p* < 0.001, respectively), although CRP remained markedly increased above the reference interval. In the hypovolaemic and dehydrated subgroups, both the HCT (*p* < 0.001 and *p* = 0.003, respectively) and CRP (*p* = 0.026 and *p* = 0.006, respectively) decreased significantly. Antithrombin activity decreased significantly in the hypovolaemic group (*p* = 0.007), yet increased significantly in the dehydrated group (*p* = 0.045). The fibrinogen concentration remained above the assay detection limit. No significant changes were seen in the CRI group, or the hypovolaemic and dehydrated subgroups (Table 3).

### Correlation analysis

For the CPV group at admission, R had a moderate negative correlation with AT activity (r_s_ = −0.405, *p* = 0.036); and MA a moderate positive correlation with the platelet count (r_s_ = 0.464, *p* = 0.015). Within the dehydrated subgroup at admission, AT activity was strongly correlated with the α-angle (r_s_ = 0.691, *p* = 0.019).

Post fluid resuscitation, MA retained a moderate positive correlation with platelet count (r_s_ = 0.448, *p* = 0.019) in the CPV group. Antithrombin activity had a moderate negative correlation to the change in R (r_s_ = −0.430, *p* = 0.025). In addition, the volume of fluid administered had a strong positive correlation with the decline in AT activity (r_s_ = 0.726, *p* < 0.001). The change in CRP had a moderate positive correlation with R (r_s_ = 0.452, *p* = 0.02) and K (r_s_ = 0.528, *p* = 0.006) and a moderate negative correlation with α-angle (r_s_ = −0.461, *p* = 0.018). In the hypovolaemic group, MA retained a strong positive correlation with the platelet count (r_s_ = 0.656, *p* = 0.006), while the volume of fluids administered had a moderate positive correlation with the decline in AT activity (r_s_ = 0.521, *p* = 0.046) and a moderate negative correlation with the platelet count (r_s_ = −0.538, *p* = 0.039). In the dehydrated group, MA retained a strong positive correlation with the platelet count (r_s_ = 0.648, *p* = 0.031) and the volume of fluid administered had a strong positive correlation to the change in AT activity from admission (r_s_ = 0.843, *p* = 0.001).

## Discussion

The haemostatic status of dogs hospitalised with CPV enteritis at admission and after fluid resuscitation in this study was characterised by an increased clot strength (MA) and delayed clot initiation (R, K, α-angle) compared to healthy control dogs. The MA increased, clot initiation time shortened and AT activity decreased after crystalloid fluid resuscitation in dogs that received large volumes of fluids. The increased MA and decreased AT activity persisted up to 3 h after resuscitation with crystalloid fluid therapy, while still receiving a constant rate of fluids.

The R and K variables were significantly longer and the α-angle significantly smaller for the CPV group at admission, indicating delayed clot initiation and amplification compared to the healthy control group. The prolonged R and K were also present in the hypovolaemic subgroup at admission compared to the controls, but not in the dehydrated subgroup. The R, K and α-angle variables measure the time to, and rate of, clot generation (Donahue & Otto 2005). The R is affected by clotting factors whereas K and α-angle are determined by platelet count and function, thrombin formation, fibrin precipitation, fibrinogen concentration and HCT, in addition to clotting factors (Donahue & Otto 2005). Prolonged clot initiation was not reported previously in dogs with CPV enteritis (Otto et al. 2000); however, activated partial thromboplastin time was moderately prolonged, possibly indicating clotting factor deficiency of the intrinsic pathway. The prolonged R in the CPV group may be explained by the higher HCT of these dogs compared to the controls. Plasma coagulation proteins are distributed in the liquid component of blood, thus blood with a higher HCT contains fewer plasma proteins and subsequent interactions per volume of blood sample (Smith et al. 2012). Delayed clot initiation with increasing HCT has been reported in humans without disease (Nagler et al. 2013). The reason for the prolonged R in the hypovolaemic subgroup, but not the dehydrated subgroup, compared to the controls, is unclear. Higher HCT could not be argued as the reason, because there was no significant difference in HCT between the hypovolaemic and dehydrated subgroups. In contrast, K and α-angle and the factors known to influence them were disparate between the CPV and control groups. For example, while the higher HCT in the CPV group would contribute to a longer K and smaller α-angle, the higher fibrinogen concentration in these dogs should contribute to a shorter K and larger α-angle. The reason for the prolonged K in the hypovolaemic subgroup only is unclear and could be the result of low case numbers. The factors other than platelet count, HCT and fibrinogen concentration that may influence K and α-angle, namely factor II, factor VII, thrombin formation and fibrin precipitation (Donahue & Otto 2005), were not evaluated in this study.

Maximum amplitude reflects maximal clot strength and is influenced by the fibrin and fibrinogen concentration, platelet count, platelet function, thrombin concentration, factor XIII and HCT (Donahue & Otto 2005). The MA in the CPV group at admission was significantly higher compared to the controls. This is similar to a previous study that found a higher MA in CPV dogs within 24 h of admission after fluid administration compared to the controls (Otto et al. 2000). The increased MA may be explained by the marked inflammation present in dogs with CPV enteritis (McClure et al. 2013) and is supported in this study by the significantly elevated CRP and fibrinogen concentrations in this group compared to the controls. A more hypocoagulable TEG tracing would have been expected in the CPV group, considering the higher HCT (Bochsen et al. 2011; Nagler et al. 2013; Smith et al. 2012); however, the effect of HCT may have been masked by the overwhelming inflammatory response and subsequent activation of haemostasis. Inflammatory cytokines such as tumour necrosis factor, interleukin-6 and interleukin-1 have a direct effect on the vascular endothelium, including induction of tissue factor expression, alteration in the thrombogenicity of endothelial surfaces, and platelet activation and adhesion to endothelial cells (Weiss & Rashid 1998). Platelet count was similar between the CPV and control groups and thus did not contribute to the difference in MA between groups. Other factors known to affect MA, such as platelet function, fibrin and thrombin concentration, were not measured. The lack of any significant difference between the hypovolaemic and dehydrated subgroups at admission allowed for comparison of the effect of the volume of fluids administered between the two subgroups.

The changes noted in the CPV group of significantly shorter clot initiation (shorter R and K, and larger α-angle) and increased clot strength (larger MA) after fluid resuscitation compared to admission may be as a result of the crystalloid fluid therapy, the progression of disease or a combination of these two factors. In the CPV group, the magnitude of decrease in HCT after fluid resuscitation correlated moderately with the volume of fluid administered, as would be expected. The significant decrease in HCT after fluid therapy may have contributed to the increased MA, as reported in an *in vivo* study in dogs that demonstrated increased MA secondary to a transient decrease in red cell mass after whole blood replacement with plasma (McMichael et al. 2014). Possible explanations for increased clot strength associated with *in vitro* anaemia models include a decreased citrate to plasma protein ratio, increased contact activation of anaemic blood with the TEG cup, or decreased red blood cell mass that allows for a tighter fibrin meshwork (Bochsen et al. 2011; Brooks et al. 2014; McMichael et al. 2011). Furthermore, the ongoing and progressive inflammatory process resulting in persistently high fibrinogen concentrations, together with increased platelet count (the significance of which is unclear) and possible platelet activation, may have contributed to increased clot strength. Moreover, reperfusion after fluid therapy has been reported to result in the release of inflammatory mediators and tissue factor, with subsequent coagulation cascade activation (Krentz & Allen 2017). The surface layer of the vascular endothelium, the endothelial glycocalyx, is rich in proteins, proteoglycans and glycosaminoglycans. In a canine haemorrhagic shock model, large-volume crystalloid fluid resuscitation has been shown to result in hyaluronan shedding from the endothelial glycocalyx. Hyaluronan can stimulate chemokine and cytokine production and promote systemic inflammation (Smart et al. 2018).

Within the subgroups, MA only increased significantly in the hypovolaemic group, while the R, K and α-angle did not change significantly for either subgroup. It is possible that the dehydrated group did not receive enough fluids to affect haemostasis, particularly MA, as these dogs received only 90 min – 120 min of a 24-h rehydration fluid plan. However, the volume of fluids administered was not correlated to the change in MA in the CPV group or either subgroup. The median volume of LRS received in the hypovolaemic group approached the 33% *in vitro* dilution rate of 33 mL/kg in the study by Morris and co-workers that reported the prolongation of K, reduced α-angle and decreased MA (Morris et al. 2016). The changes in the CPV group and subgroups did not mimic the findings of previously described *in vitro* canine studies in healthy dogs (Falco et al. 2012; Morris et al. 2016). The results of *in vitro* studies do not necessarily translate to a clinically relevant scenario because of the absence of blood flow, endothelium, natural metabolic degradation and compensatory mechanisms (Falco et al. 2012; Morris et al. 2016). In one study using rabbits, TEG was performed after both *in vivo* and *in vitro* haemodilution, and a poor correlation was found for all variables, except MA, showing weak agreement (Nielsen & Baird 2000).

The change in AT activity from admission to the end of fluid resuscitation had a strong correlation with the volume of fluid administered in the CPV group, as well as a strong correlation in the dehydrated subgroup and a moderate correlation in the hypovolaemic subgroup. A possible explanation for the correlation between the magnitude of reduction in AT activity and the volume of fluid administered could be that the reduction of AT activity after fluid therapy may, in part, be due to haemodilution. An *in vitro* study in humans reported that up to 30% dilution of plasma resulted in shortened R, with the same effect not evident if AT-deficient plasma was diluted (Nielsen, Lyerly & Gurley 2004). That study suggests that the dilution of AT may cause a shortened clot-initiation time after fluid therapy, but celite was used as an activator in that study and the findings cannot necessarily be extrapolated to tissue factor-activated TEG. The change in AT activity after fluid resuscitation might explain the changes that were seen in TEG variables after fluid resuscitation, such as the shortened R. The reduced AT activity in our study may also have been the result of a number of other factors including the increased loss of AT as a result of improved perfusion of the gastrointestinal tract after fluid resuscitation, severe protein-losing enteropathy, dilution and acidification of blood secondary to LRS infusion, AT consumption as a result of the activation of coagulation, and disease progression (Brandtzaeg et al. 1989; Gissel et al. 2016; Otto et al. 2000). A recent study also demonstrated that dogs with CPV have evidence of acute kidney injury and significantly increased urine protein to creatinine ratios compared to healthy control dogs (Van den Berg et al. 2018). Thus, a protein-losing nephropathy could also contribute to the loss of AT in these dogs. Antithrombin concentration, thrombin to AT complexes and changes in acid-base status were not measured in this study and require further investigation. The unexpected increase of AT activity in the dehydrated subgroup is unclear and contradictory to what would be expected in parvo-infected dogs with a protein-losing nephropathy.

The lack of significant changes in haemostatic variables after 3 h of approximate maintenance-rate fluids in the CPV group, including subgroups, suggests that the shortened clot initiation time and increase in clot strength after fluid resuscitation is not transient and is unlikely to be exclusively a result of the effect of dilution by fluids.

Further investigation is required to determine the clinical relevance of a hypercoagulable state in dogs with CPV enteritis. The effect of crystalloids on TEG is significantly less than the effect of colloids in dogs with inflammation (Gauthier et al. 2014). Further studies are warranted to evaluate the effect of natural and synthetic colloids in dogs with CPV enteritis. Fresh frozen plasma transfusions have shown promise in reducing clinical signs and morbidity in dogs with CPV (Goddard & Leisewitz 2010; Ishibashi et al. 1983; Nguyen et al. 2006), but could possibly worsen the hypercoagulable state that is present in these dogs by increasing the fibrinogen concentration and other coagulation factors (Otto et al. 2000). Acute thromboembolic events have been anecdotally reported as a cause of acute death, but studies have not investigated the occurrence of thrombi or thromboemboli in dogs that die of CPV enteritis. It is also unknown whether or not specific therapy is indicated to prevent thromboembolic complications. The increase in clot strength and faster clot initiation after fluid therapy also requires further investigation in other diseases, to determine its clinical relevance.

Several limitations inherent to clinical studies were present in this study. Disease severity and duration of illness could not be standardised. The determination of illness severity scores and severity prediction index would have allowed a more accurate comparison between the subgroups of dogs with CPV. All dogs diagnosed with CPV enteritis at the OVAH are offered hospitalisation and treatment, regardless of the severity of illness, and although a bias towards sicker dogs in this study is likely, it cannot be confirmed. The findings of the study can therefore not be extrapolated to dogs with milder disease treated on an outpatient basis. The extent of dehydration was estimated based on subjective guidelines. The relatively small sample size may have affected the statistical analysis, particularly in the CRI group. There were small variations in the time (5 min – 15 min) from admission blood sampling to the initiation of fluid therapy, the speed at which fluid boluses were given and the time between fluid boluses, although all of these were case-appropriate. The objective of the study was to determine haemostatic changes after fluid resuscitation, and the true effect of the minor time differences could not be determined from the study. The lack of a CPV group that did not receive fluids did not allow for determination of the cause of the increase in the hypercoagulable state after fluid administration, but would not have been ethically acceptable. The measurement of the D-dimer concentration and fibrin degradation products were not performed in this study. No measurable D-dimer concentrations were found in a previous study evaluating TEG in CPV (Otto et al. 2000). The measurement of additional coagulation tests, such as prothrombin time, activated partial thromboplastin time, platelet function testing, thrombin concentration, thrombin to AT complexes and individual coagulation factor testing was not performed and would have allowed more evaluation of the haemostatic status in dogs with CPV as TEG provides complementary, but not interchangeable, information to the traditional coagulation tests (Rubanick et al. 2017).

## Conclusion

In conclusion, this study showed that although clot initiation is delayed at admission in dogs hospitalised with CPV enteritis, clot strength is increased compared to healthy control dogs. Crystalloid fluid resuscitation resulted in shortened clot initiation (shorter R and K, and larger α-angle) and further increased clot strength, as evidenced by larger MA in dogs with CPV that were hypovolaemic at presentation. Increased clot strength persisted for hours after large-volume crystalloid fluid administration.
